# Empathy and Parental Sensitivity in Child Attachment and Socioemotional Development: A Systematic Review from Emotional, Genetic, and Neurobiological Perspectives

**DOI:** 10.3390/children12040465

**Published:** 2025-04-04

**Authors:** Miriam Santana-Ferrándiz, Jesús Ibáñez-Pérez, Carmen Moret-Tatay

**Affiliations:** 1 Doctoral School, Catholic University of Valencia San Vicente Mártir, 46002 Valencia, Spain; 2 Faculty of Psychology, Catholic University of Valencia San Vicente Mártir, 46100 Valencia, Spain; jesus.ibanez@ucv.es (J.I.-P.); mariacarmen.moret@ucv.es (C.M.-T.)

**Keywords:** parental empathy, parental sensitivity, child attachment, socioemotional development, emotional regulation, genetic predispositions, neurobiological mechanisms, systematic review

## Abstract

Parental empathy and sensitivity play a crucial role in the development of child attachment and socioemotional growth, influencing emotional regulation, social skills, and psychological well-being. However, no comprehensive systematic review integrates emotional, genetic, and neurobiological perspectives. Objectives: this systematic review aims to synthesize existing evidence on the relationship between parental empathy and sensitivity with child attachment and socioemotional development, integrating classical theories with contemporary findings and considering contextual factors such as adversity and intergenerational dynamics. Method: searches were conducted for studies published between 1993 and 2024. Empirical studies examining empathy (affective, cognitive, and multidimensional), parental sensitivity, child attachment (secure, avoidant, ambivalent, disorganized, or DMM), and socioemotional development were included. A total of 16 studies met the inclusion criteria, encompassing longitudinal, cross-sectional, genetic, neurobiological, and experimental designs. Results: key findings include the following: positive socialization predicted greater empathy, and self-regulation maternal anxiety reduced sensitivity and indirectly affected attachment; emotional empathy positively influenced sensitivity; genetic predispositions affected sensitivity through crying; neurobiological studies revealed altered PCC–amygdala connectivity in postpartum depression. Conclusions: the findings demonstrate that parental empathy and sensitivity significantly influence child attachment security and socioemotional development through emotional regulation, genetic predispositions, and neurobiological mechanisms. This review provides a comprehensive framework for understanding the parent–child bond and highlights implications for evidence-based parenting interventions.

## 1. Introduction

In the mid-20th century, British psychiatrist and psychoanalyst John Bowlby, influenced by evolutionary theories, developed attachment theory, defining it as an adaptive behavioral system arising from the human need to establish emotional bonds with caregivers, thereby ensuring survival and emotional development [1]. This approach revolutionized the understanding of early relationships by proposing that attachment is not solely based on satisfying basic needs, such as food, but on the need for proximity and emotional security. The quality of this bond significantly influences the child’s emotional regulation, environmental exploration, and interpersonal relationships. Bowlby’s work was complemented by Mary Ainsworth, who developed the Strange Situation Procedure, identifying patterns of secure, insecure-ambivalent, insecure-avoidant, and later, disorganized attachment [2,3]. Ainsworth also introduced the construct of parental sensitivity, defined as caregivers’ ability to interpret and respond appropriately to their children’s signals. Parental sensitivity provides a “secure base” that fosters the child’s exploration and self-regulation, establishing a critical foundation for secure attachment.

Beyond the traditional model centered on sensitivity, alternative theoretical approaches have emerged to explain how attachment may vary according to the context. In this regard, psychologist Patricia Crittenden proposed the Dynamic-Maturational Model of Attachment (DMM), which expands Ainsworth’s classical model. This approach considers attachment patterns as adaptive strategies developed in response to specific contexts, particularly under conditions of stress or danger, thereby broadening the understanding of attachment in different environments [4,5].

Contemporary research highlights parental empathy as a mediator of parental sensitivity, emphasizing empathy’s emotional and cognitive dimensions [6,7]. Parental empathy, defined as the capacity to resonate with the child’s emotional states and respond with understanding and care, is linked to neurobiological mechanisms that promote consistent and warm interactions, which are crucial for developing secure attachment. Neuroimaging studies have identified brain networks related to embodied simulations and mentalization, underlying parental empathy and contributing to child emotional regulation [6,8]. Davis’s model [9] provides a useful framework for understanding this multidimensional empathy, distinguishing affective and cognitive components across four main dimensions: perspective-taking, the cognitive ability to adopt another person’s point of view; empathic concern, the emotional response oriented towards another’s well-being; personal distress, the negative emotional reaction when witnessing others’ suffering; and fantasy, the ability to imagine oneself in fictional situations. These dimensions enable a nuanced understanding of empathy’s role in children’s socioemotional competence [10,11].

Moreover, recent studies have explored factors influencing empathy and sensitivity, such as parental post-traumatic stress and intergenerational transmission of attachment styles [10]. In contexts of adversity, such as poverty, forced migration, or exposure to violence, parental sensitivity can diminish, adversely impacting child socioemotional development [12,13]. For example, chronic poverty can elevate caregiver stress, reducing sensitivity toward children’s emotional needs [14], while trauma related to forced migration may compromise secure attachment formation [15].

On the other hand, Crittenden’s Dynamic-Maturational Model of Attachment (DMM) has expanded the traditional model by emphasizing that parental sensitivity is a dynamic construct influenced by contextual factors such as trauma, stress, and caregivers’ attachment experiences [4,5]. This model suggests that in adverse contexts, caregivers may develop adaptive attachment strategies that, while functional in the short term, can limit their ability to provide consistent parental sensitivity. For example, a caregiver who has experienced trauma may display disorganized attachment patterns, hindering their ability to interpret and respond appropriately to their child’s emotional signals [4].

Addressing this gap, this study systematically reviews how parental empathy and sensitivity influence child attachment and socio-emotional, integrating emotional, genetic, and neurobiological perspectives, following PRISMA guidelines [16], and covering research published from 1993 to 2024.

## 2. Materials and Methods

This study is a systematic review conducted following the PRISMA 2020 guidelines [16], aiming to synthesize the available evidence on empathy and parental sensitivity in relation to child attachment and socioemotional development.

### 2.1. Search Strategy

The following EBSCOhost databases were included in the search: (i) PsycINFO, (ii) MEDLINE, (iii) Academic Search Complete, (iv) Communication & Mass Media Complete, (v) ERIC, (vi) SocINDEX, and the (vii) Film & Television Literature Index. The search was limited to studies published between 1993 and 2024 in English. The search was performed using the following Boolean search syntax:

*(maternal sensitivity or parental sensitivity or caregiver sensitivity) AND (attachment theory or attachment or attachment style) AND empathy*

### 2.2. Eligibility Criteria

Empirical studies (quantitative, qualitative, or mixed methods) examining the relationship between empathy and/or parental sensitivity and child attachment or socioemotional development were included. Participants had to be children aged from 0 to 12 years and their primary caregivers. The variables of interest included parental empathy (affective, cognitive, or multidimensional), parental sensitivity, child attachment (secure, avoidant, ambivalent, and disorganized), and socioemotional development (emotional self-regulation, social skills, and psychological well-being). Studies conducted in everyday settings and contexts of adversity with emotional, contextual, genetic, and neurobiological approaches were considered.

Studies were excluded if they were theoretical papers, narrative reviews, case studies, or anecdotal reports. Studies on adolescents or adults without a retrospective analysis of childhood attachment were also excluded, as well as studies on secondary caregivers unless they were the primary attachment figure. Additionally, studies that did not explicitly relate parental empathy or sensitivity to child attachment or socioemotional development were excluded. Studies published before 1993 were excluded, as from that date onwards, research on attachment has incorporated significant advances in neurobiology, genetics, and dynamic attachment models, ensuring that this review is based on an updated theoretical framework (except for foundational studies), and articles in languages other than English, or without full-text access, were also excluded.

### 2.3. Study Selection

Two independent reviewers screened the results using the Rayyan web-based platform (QCRI) to enhance the rigor and efficiency of the systematic review. In cases of disagreement regarding the inclusion of a study (four conflicting cases), a third independent reviewer was consulted to make the final decision. To ensure consistency, both reviewers participated in a calibration exercise prior to screening, where they independently assessed a subset of 15 articles and discussed discrepancies to align their understanding of the inclusion criteria.

### 2.4. Data Extraction and Quality Assessment

A standardized form was developed for data extraction, including information about the author, year of publication, study design, sample characteristics, measures used, and main findings. The methodological quality of the included studies was assessed using a modified version of the Newcastle–Ottawa scale (NOS) for observational research. Studies were evaluated based on three main domains: (1) selection of study groups (e.g., representativeness of the sample and adequacy of the sample size), (2) comparability of groups (e.g., control for confounding factors), and (3) assessment of outcomes (e.g., validity and reliability of measures). A predefined threshold of 6 out of 9 points was used to classify studies as having moderate-to-high quality, ensuring that only robust evidence was included in the final synthesis.

Given the diversity of methods used to assess parental sensitivity (e.g., Ainsworth’s Maternal Sensitivity Scale, fMRI, and self-reports), we categorized findings based on the type of measure and discussed potential measurement errors and limitations in the interpretation of results. For example, self-report measures may be subject to social desirability bias, while observational scales provide more objective but context-specific data.

## 3. Results

A systematic approach was used to identify and screen relevant studies for inclusion. Table 1 summarizes the characteristics of the included studies. The studies varied in design, sample size, and context, but they all examined the relationship between parental empathy and/or sensitivity and child attachment or socioemotional development.

Lastly, the risk of bias assessment (Table 2) showed that the majority of longitudinal studies, particularly those with robust genetic or neurobiological assessments and well-characterized samples, had a low risk of bias. In contrast, six studies were found to have a moderate risk of bias, predominantly in cross-sectional and experimental studies with small sample sizes, which limits causal inferences.

**Figure 1:**
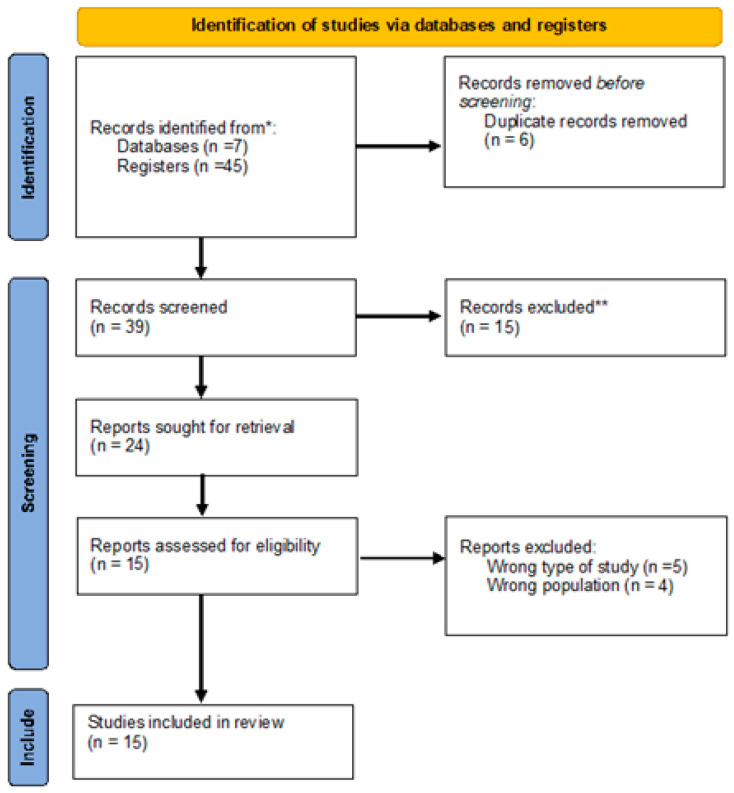
PRISMA flow diagram of the study selection process for the current systematic review. Notes: * Records identified from Rayyan following EBSCOhost databases; ** excluded in terms of exclusion criteria applied to title and abstract.

Figure 1 presents the PRISMA flow diagram, which outlines the different phases of the study selection process, including identification, screening, eligibility assessment, and final inclusion.

## 4. Discussion

The findings of this systematic review confirm that parental sensitivity is a key predictor of secure attachment and child socioemotional development. However, this relationship is neither unidirectional nor static; rather, it is shaped by a complex interaction of emotional, genetic, and neurobiological factors that influence caregiving behaviors and attachment security. The reviewed studies demonstrate that both maternal and paternal sensitivity contribute significantly to the child’s ability to regulate emotions, develop social competence, and form stable interpersonal relationships [17,27,28].

Empirical evidence supports the notion that consistent and emotionally attuned caregiving fosters secure internal models in children, which in turn promote greater emotional regulation and empathy [17,19,20]. Conversely, inconsistent or insensitive caregiving is associated with insecure attachment styles, leading to difficulties in socioemotional adjustment [18,24,29]. In a notable study, infants with less secure attachment (and temperamental indicators of higher fear) exhibited reduced empathy toward the distress of others, while securely attached children demonstrated higher empathic concern and responsiveness [31].

Genetic and environmental factors further moderate these relationships. Research suggests that maternal anxiety, parental rigidity, and genetic markers such as AVPR1a and DRD4 influence sensitivity, making some parents more predisposed to responsive caregiving, while others may struggle, particularly in stressful situations [23,26,28,30]. The interaction between genetics and parenting style highlights the importance of a bidirectional influence in attachment formation. Studies indicate that children with certain genetic variants are more sensitive to the quality of caregiving, meaning that they benefit significantly from high parental sensitivity but may be disproportionately affected by insensitive parenting [23].

Neurobiological findings support these behavioral observations by showing that parental sensitivity is linked to distinct brain activations. Parents exhibiting higher sensitivity show increased activity in prefrontal areas associated with empathy and regulation, as well as in limbic regions involved in emotional processing and bonding [19,24,26]. Conversely, disruptions in these circuits, such as those seen in postpartum depression, correlate with lower responsiveness to infant cues, suggesting that neural pathways play a critical role in caregiving behaviors [19,22]. Moreover, oxytocin release in response to infant interaction has been shown to reinforce bonding and reduce parental stress, further strengthening the neurobiological underpinnings of attachment formation [21].

Affective sensitivity, or the ability of caregivers to emotionally attune to their children, emerges as another crucial mechanism underpinning attachment security. Research suggests that the ability of caregivers to regulate their own emotional responses plays a significant role in establishing affective sensitivity in interactions with their children, particularly in moments of distress [22,27]. Caregivers who display higher emotional regulation are more likely to provide consistent and supportive responses, reinforcing secure attachment patterns. Studies highlight that dyads characterized by secure attachment show greater affective sensitivity and mutual understanding, especially in stressful situations [28]. This emotional co-regulation not only strengthens the attachment bond but also facilitates the development of socioemotional competencies such as perspective-taking and empathic concern [20,28]. Additionally, studies indicate that disruptions in affective sensitivity, whether due to parental stress or external environmental factors, can contribute to inconsistencies in attachment security and limit a child’s ability to develop effective socioemotional skills [27,30].

### 4.1. Limitations

The limitations of this review primarily concern methodological heterogeneity across the included studies. Several investigations use cross-sectional designs, which make it difficult to establish causal relationships [19,26]. Likewise, some studies rely on small or highly specific samples, for instance, mothers of premature infants [18] or mothers with postpartum depression [19] thus restricting the generalizability of the findings. Furthermore, the methods used to measure sensitivity and empathy vary from self-report questionnaires to direct observations [17,22], increasing the risk of social desirability bias and complicating direct comparisons across studies. Finally, in those works with genetic or neurobiological components, the use of correlational designs and small sample sizes demands caution when interpreting the associations between biological factors and parenting behavior [23,26].

### 4.2. Practical Implications and Intervention Recommendations

Given the well-documented impact of parental sensitivity on attachment and socioemotional development, targeted interventions should focus on strengthening caregivers’ ability to respond appropriately to their children’s emotional needs. Parenting programs that emphasize emotional regulation, mentalization, and affective sensitivity have been shown to improve parental sensitivity and attachment outcomes [25,30].

One promising avenue involves integrating neurobiological and genetic insights into intervention strategies. For instance, parents with a predisposition toward lower sensitivity (due to genetic markers or past attachment experiences) could benefit from tailored support that enhances their ability to engage in emotionally attuned caregiving. Similarly, programs targeting high-risk groups, such as parents experiencing postpartum depression or chronic stress, should incorporate techniques designed to mitigate the neurobiological disruptions that impact caregiving behaviors [17,30].

Education professionals and early childhood caregivers could also benefit from these findings by adopting attachment-based frameworks in classroom environments. Secure teacher–child relationships have been linked to improved emotional regulation and social skills in children, mirroring the benefits of secure parental attachment [21,25]. Training educators in strategies that promote emotional attunement and responsiveness could provide additional support for children who may lack secure attachment relationships at home.

Another important area for future exploration involves cultural and societal influences on parental sensitivity. Research suggests that cultural values influence how parental sensitivity is expressed. In more individualistic societies, sensitivity may emphasize fostering independence and autonomy, whereas in collectivist cultures, it may focus on interdependence and communal caregiving practices. These variations affect the way attachment security is developed and should be considered in future interventions [25,30]. While attachment principles are universally relevant, cultural variations in caregiving practices may shape how sensitivity is expressed and interpreted. Future research should investigate how cultural norms interact with biological and emotional determinants of attachment, ensuring that interventions are culturally adaptive and accessible across different contexts [30].

## 5. Conclusions

Parental sensitivity is a multidimensional construct shaped by the intricate interplay of emotional, genetic, and neurobiological factors. Empirical evidence suggests that parental empathy is a crucial driver of sensitivity, as it enables caregivers to attune to their child’s emotional states and respond appropriately. Studies indicate that parents with higher dispositional empathy exhibit greater affective sensitivity, leading to more secure attachment bonds [17,21]. Empathy and affective sensitivity play central roles in fostering secure attachment, supported by brain mechanisms that enhance social bonding and stress regulation [19,26]. Parental empathy not only facilitates responsiveness but also reinforces emotional co-regulation between caregiver and child, promoting long-term socioemotional development [20,25,28]. The reviewed studies demonstrate that higher parental sensitivity is associated with increased neural activation in key regulatory regions, higher oxytocin levels, and more adaptive socioemotional outcomes in children [19,22]. Conversely, disruptions in these processes, such as those linked to anxiety, depression, or genetic predispositions, may negatively affect caregiver responsiveness and attachment security [23,30].

These findings underscore the importance of adopting a holistic perspective on attachment, one that incorporates insights from emotional, genetic, and neurobiological research. By understanding how these factors interact, future interventions can be designed to enhance parental sensitivity, thereby promoting healthier attachment relationships and better socioemotional development in children. Further research should continue to explore these intersections, particularly in diverse cultural and socioeconomic contexts, to refine and expand intervention strategies that support families across various backgrounds.

## Figures and Tables

**Table 1:** Summary of studies included.

Study	Design	Sample	Measures	Key Findings
Borelli et al. [17] (2016)	Cross-sectional correlational	60 dyads (USA)	Parental development interview (PDI-R-SC); PACES (Parental Affective and Cognitive Empathy Scale); dispositional empathy questionnaire (self-report by the parent); child attachment interview (CAI); child perception of parental warmth (derived from CAI); caregiver’s attachment style: adult self-report.	Parental empathy correlated with attachment security (r = 0.42, *p* < 0.01) and emotional openness (r = 0.38, *p* < 0.05).
Butcher et al. [18] (1993)	Longitudinal	Mothers of premature infants (unspecified)	Maternal rigidity, sensitivity, attachment, and infant responsiveness.	Maternal rigidity predicted lower sensitivity and insecure attachment (*p* < 0.05).
Chase et al. [19] (2014)	Cross-sectional Neurobiological	37 mothers (USA)	Neural connectivity (fMRI), Hamilton depression rating scale (HDRS), Edinburgh postnatal depression Scale (EPDS), and parent-to-infant attachment questionnaire.	Mothers with postpartum depression exhibited abnormal connectivity between the PCC (central region of the default mode network) and the right amygdala; in the depressed group, the PCC–amygdala showed negative coupling (inverse correlation), while this inverse coupling was not observed in healthy mothers. PCC–amygdala connectivity was positively correlated with PCC–parahippocampal connectivity in the overall group.
Javakhishvili and Vazsonyi [20] (2022)	Longitudinal	1364 children and their families (USA)	Maternal sensitivity; HOME inventory; strange situation; EITQ; WASI; SSRS; youth psychopathic trait inventory; self-reported delinquency.	Early positive socialization predicted higher empathy (β = 0.15) and self-control (β = 0.25) and lower emotional insensitivity (β = −0.16, *p* = 0.038) and delinquency (β = −0.22, *p* = 0.041).
Kaźmierczak et al. [21] (2024)	Experimental	221 heterosexual couples	Empathy (IRI); sensitivity; cry response (Ainsworth’s sensitivity).	Higher dispositional empathy was significantly associated with greater sensitivity in infant care (r = 0.48, *p* < 0.001). High personal distress was negatively correlated with sensitivity (r = −35, *p* < 0.05).
Leerkes [22] (2010)	Longitudinal correlational	101 mother-infant dyads (USA)	Maternal emotional reactions to crying questionnaire, emotional goal interview related to crying, maternal self-efficacy scale (adapted), CES-D (Center for Epidemiologic Studies Depression Scale), and infant distress detection measure (using video clips).	Maternal sensitivity correlated with empathy towards infant crying (r = 0.32, *p* < 0.05). High empathy and infant-oriented goals buffer the negative impact of infant distress on maternal sensitivity.
Leerkes et al. [23] (2017)	Longitudinal and genetic	207 mothers (USA)	Maternal genotypes (vasopressin receptor AVPR1a and dopamine receptor DRD4 (long vs. short allele), cry processing, Ainsworth’s Maternal Sensitivity Scale, and adult attachment interview (AAI).	Infant-oriented processing was associated with greater maternal sensitivity: B = 0.26, SE = 0.11, β = 0.13, and *p* < 0.05. Coherence of mind and maternal education were significant predictors of greater maternal sensitivity. AVPR1a and DRD4 were associated with a greater focus on the mother’s own needs, which in turn reduced maternal sensitivity.
Ma et al. [24] (2017)	Experimental cross-sectional	65 women (China)	Attachment styles (ECR); brain activity (ERP); emotional processing.	N170: no significant differences in amplitude by attachment style. P300: Securely attached women showed higher P300 amplitudes for infant faces (~7.6 µV vs. ~5.3 µV in avoidant; *p* ≈ 0.04). In response to infant crying, both secure and anxious groups had higher P300 amplitudes than the avoidant group (*p* = 0.018–0.005; *p* = 0.013). Avoidant women had the lowest P300 responses, showing minimal differentiation between crying and neutral faces, suggesting reduced engagement with infant cues.
Marakovitz [25] (2001)	Longitudinal	111 dyads (USA)	Child empathy: Evaluated 24 and 36 months in the laboratory through “harm simulation” scenarios; child temperament; maternal sensitivity: Observed in semi-structured interactions at 6, 15, and 24 months and rated on scales (e.g., Ainsworth sensitivity); strange situation.	Maternal sensitivity correlated with empathic concern (r = 0.24, *p* < 0.01).
Musser et al. [26] (2012)	Cross-sectional correlational	22 mothers (USA)	Maternal sensitivity; brain activity (fMRI); intrusiveness; dyadic harmony.	Maternal sensitivity correlated with prefrontal activation (*p* < 0.05), specifically the right frontal pole and right inferior frontal gyrus (orbitofrontal/inferior cortex). Conversely, mothers with more intrusive behavior showed significantly greater activation in the left anterior insula and temporal pole when hearing their own infant’s cry.
Nieri [27] (2017)	Cross-sectional	118 fathers (Argentina)	Paternal sensitivity questionnaire, big five inventory (BFI)reactive interpersonal index (IRI; Spanish adaptation)relationship questionnaire (Spanish adaptation).	Paternal sensitivity correlated with secure attachment (r = 0.25, *p* < 0.01). For fathers aged 18–25, avoidant attachment was negatively correlated with sensitivity (r = −0.84, *p* < 0.01). Personal distress was linked to lower empathy in negative situations (r = −0.43, *p* < 0.01). For fathers aged 31–50, secure attachment was weakly correlated with sensitivity (r = 0.21, *p* < 0.05). Higher sensitivity was related to greater empathy, especially in perspective-taking and lower personal distress. Fathers in stable relationships showed higher sensitivity, empathy, and secure attachment, while separated fathers tended to develop fearful behaviors.
Pederson et al. [28] (2014)	Longitudinal	64 mother–infant dyads (Canada)	Maternal sensitivity (Q-set); infant attachment (strange situation); relational harmony.	Maternal sensitivity correlated with infant attachment (r = 0.45, *p* < 0.001).
Pillai et al. [29] (2011)	Longitudinal	731 dyads (Canada)	Caregiver sensitivity (emotional availability scales); infant pain behavior (NFCS).	Caregiver sensitivity showed significant stability over time: Sensitivity at 2, 4, 6, and 12 months was autocorrelated (e.g., 6→12 months β = 0.62, *p* < 0.001). At 12 months, a significant association emerged: more sensitive caregivers had infants with fewer prolonged pain expressions after vaccination (residual correlation with NFCS at 1 min: r = −0.39, *p* < 0.001). At 2, 4, and 6 months, the concurrent correlation between sensitivity and pain response was not significant.
Stevenson-Hinde et al. [30] (2013)	Cross-sectional	98 mothers (UK/Netherlands)	Maternal anxiety (HADS); maternal sensitivity; infant attachment (modified strange situation).	Maternal anxiety reduced sensitivity (β = −0.34) and indirectly affected attachment. Sensitivity explained 33% of attachment security variance (β = 0.58, *p* < 0.001).
Van der Mark et al. [31] (2002)	Longitudinal	125 girls and their mothers (Netherlands)	Child empathy (observed); child temperament (fear); maternal sensitivity; strange situation; Bayley scales of infant development.	Attachment security and maternal sensitivity did not show significant correlations with empathy in most cases. In regression analyses, low attachment security (β ≈ 0.19, *p* = 0.04) and higher child fear (β ≈ −0.20, *p* = 0.02) predicted lower empathy toward the stranger at 22 months. Paradoxically, higher concurrent maternal sensitivity was associated with lower child empathy toward the stranger (r = −0.24, *p* < 0.01).

**Table 2:** Risk of bias.

Study	Design	Risk of Bias
Borelli et al. [17] (2016)	Cross-sectional and Experimental	Moderate risk: Cross-sectional design limits causal inferences.
Butcher et al. [18] (1993)	Longitudinal	Low risk: Longitudinal design, well-characterized sample.
Chase et al. [19] (2014)	Cross-sectional and Neurobiological	Moderate risk: Small sample, cross-sectional design.
Javakhishvili and Vazsonyi [20] (2022)	Longitudinal and Neurobiological	Low risk: Robust design, genetic and neurobiological evaluation.
Kaźmierczak et al. [21] (2024)	Longitudinal	Low risk: Large sample, robust design, multiple standardized measures.
Leerkes [22] (2010)	Experimental	Moderate risk: Experimental design, small sample.
Leerkes et al. [23] (2017)	Longitudinal	Low risk: Longitudinal evaluation and direct observation.
Ma et al. [24] (2017)	Longitudinal and Genetic	Low risk: Robust design, genetic evaluation, and direct observation.
Marakovitz [25] (2001)	Neurocognitive	Low risk: Experimental design, neurocognitive evaluation.
Musser et al. [26] (2012)	Longitudinal	Low risk: Well-characterized sample, longitudinal evaluation.
Nieri [27] (2017)	Neurocognitive	Moderate risk: Small sample, cross-sectional design.
Pederson et al. [28] (2014)	Cross-sectional	Moderate risk: Cross-sectional design limits causal inferences.
Pillai et al. [29] (2011)	Longitudinal	Low risk: Use of MBQS and the strange situation procedure, well-characterized sample.
Stevenson-Hinde et al. [30] (2013)	Longitudinal	Low risk: Large sample, longitudinal evaluation.
Van der Mark et al. [31] (2002)	Longitudinal	Low risk: Well-characterized sample, longitudinal evaluation.

## Data Availability

Data are contained within the article.
